# Spiking Neural Network with Linear Computational Complexity for Waveform Analysis in Amperometry

**DOI:** 10.3390/s21093276

**Published:** 2021-05-10

**Authors:** Szymon Szczęsny, Damian Huderek, Łukasz Przyborowski

**Affiliations:** Institute of Computing Science, Faculty of Computing and Telecommunications, Poznan University of Technology, Piotrowo 3A Street, 61-138 Poznań, Poland; damian.huderek@put.poznan.pl (D.H.); lukasz.i.przyborowski@doctorate.put.poznan.pl (Ł.P.)

**Keywords:** amperometry, edge computing, spiking neural network, exocytosis, vesicle fusion

## Abstract

The paper describes the architecture of a Spiking Neural Network (SNN) for time waveform analyses using edge computing. The network model was based on the principles of preprocessing signals in the diencephalon and using tonic spiking and inhibition-induced spiking models typical for the thalamus area. The research focused on a significant reduction of the complexity of the SNN algorithm by eliminating most synaptic connections and ensuring zero dispersion of weight values concerning connections between neuron layers. The paper describes a network mapping and learning algorithm, in which the number of variables in the learning process is linearly dependent on the size of the patterns. The works included testing the stability of the accuracy parameter for various network sizes. The described approach used the ability of spiking neurons to process currents of less than 100 pA, typical of amperometric techniques. An example of a practical application is an analysis of vesicle fusion signals using an amperometric system based on Carbon NanoTube (CNT) sensors. The paper concludes with a discussion of the costs of implementing the network as a semiconductor structure.

## 1. Introduction

Processing data close to its source is a frequently used approach in IoT [1]. However, performing calculations directly in sensors is a big challenge due to the need to process specific signals for the given technique. One of the most difficult techniques in this regard is amperometry—processing signals with values below 100 pA [2]. This technique is used to detect compounds such as cyanide, sulfide, sulfate, and hydrazine. Yet, it is also often used in analyzing the life processes of cells [3]. Monitoring the processes of exocytosis and endocytosis taking place in cells is possible using amperometric systems, which make it possible to analyze currents with values below 80 pA. The conversion of such signals into a digital form remains a challenge despite using ADC converters based on ΣΔ modulators and implemented using CMOS technologies and operating in the moderate-inversion mode [4]. Although circuits of such a type process currents with values below 1 nA, typical applications of amperometric techniques require processing signals an order of magnitude smaller. A certain alternative is to use the weak-inversion mode [5] in computing circuits, yet this mode limits the maximum operating frequency of converters to several hundred Hz. Alternatives to the low-voltage CMOS converters and preprocessors are hardware implementations of Spiking Neural Networks (SNNs), which are modeled on biological neurons and interpret currents in the range of 0–100 pA, generating signals with frequencies up to several dozen kHz [6]. Currently, semiconductor implementations of spiking neurons published in literature are characterized by both small dimensions at the level of single micrometers and very low power consumption at the level of a single fJ/spike [7]. However, while the parameters of individual spiking neurons implemented in CMOS technology are impressive, the parameters of entire SNNs are no longer satisfactory due to the large, often exponential complexity of neural networks and the need to use multiple layers of neurons and multiple layers of connections.

Currently, the development of the subject of artificial neural networks is dominated by deep networks. Currently, the most complex structure known to the authors is the ResNet152 network [8]. Using multilayer networks increases, on the one hand, the precision of the generated responses, but on the other hand, it leads to a significant increase in the complexity of network algorithms. Further development of this research area requires using large computing centers, which makes the developed solutions impossible to apply in practical applications [9]. The exponential complexity of neural networks and the number of connections between neurons, translating into the number of needed multiplications, are the main barriers concerning the use of neural networks in edge computing. In the IoT sector, for example, the implementation possibilities of deep neural networks are limited by the available resources of microprocessor architectures, FPGAs, and GPUs. It is worth emphasizing that deep networks are primarily strong data processing algorithms, while conceptually being quite distant from the basic assumptions of artificial intelligence. Alternatives are biologically inspired networks. Spiking neural networks are currently one of the most rapidly developed research areas in the field of artificial intelligence and cognitive science. There are high hopes concerning these networks due to their better perception properties compared to the so-called second-generation networks. For example, individual spiking neurons are capable of solving nonlinear problems [10]. Thanks to the possibility of implementing spiking neurons using silicon structures, their complex mathematical models are no longer limitations. This is because spiking neurons can be implemented using just a dozen field-effect transistors [7]. For this reason, this subject has also attracted the attention of IT companies, which offer neuroprocessors based on SNNs, e.g., the Intel-Loihi processor [11] and IBM TrueNorth processor [12]. There are many indications that a hardware implementation of spiking neurons will contribute to a significant development of this area of artificial intelligence in the near future.

This paper focused on issues concerning implementing hardware SNNs in edge computing applications. The basic area of application of such solutions is the processing (compression, classification, conversion) of signals from sensors adjacent to data sources using mobile devices. The available resources of mobile devices require using simple, but effective neural network architectures. This paper proposed an SNN with linear complexity, with a significantly reduced number of weights, which makes it possible to overcome the limitations related to the availability of multipliers, and a reduced dispersion of weights, which makes it possible to use smaller bit representations.

The paper is organized as follows. Section 2 describes a network architecture and models of the spiking neurons used. Section 3 focuses on the training of the proposed network architecture. Section 4 presents the amperometric system based on SNNs and an example of a practical application of the network in the task of classifying the patterns of current signals. The paper concludes with a brief discussion of the costs of hardware implementation, mismatch analysis, and a summary.

## 2. Network Architecture

This section describes the structure of an SNN with linear complexity. The choice of two types of neurons and their mathematical models are justified. Particular attention is paid to routing neurons.

### 2.1. Thalamo-Based Neurons

In the search for the appropriate types of spiking neurons, the authors focused on tonic spiking and inhibition-induced spiking neurons, characteristic of the thalamus area in the diencephalon [13]. The task of this area of the brain is to process most of the signals coming from all senses except smell. The thalamus preprocesses and routes sensory and movement data before sending them to other areas of the brain. The functionality of the thalamus as a sensory data preprocessor was used to develop a compact SNN dedicated to processing sensor data as part of edge computing.

Modeling included using the Izhikevich model, characterized by a complexity similar to the integrate-and-fire model and the fidelity of the mapping of a biological neuron comparable to the complex Hodgkin–Huxley model. Due to the use in edge computing applications, the key is the number of floating point operations, which for these models is as follows: integrate-and-fire, 5; Izhikevich, 13; Hodgkin–Huxley, 1200 [14]. The Izhikevich model is defined by Equations (1)–(3) [15].

(1) $$\frac{dv}{dt}=0.04v^{2}+5v+140-u+I_{app}$$

(2) $$\frac{du}{dt}=a\left(bv-u\right)$$

(3) $$\begin{array}{cccc} if & v\geq 30\;\mathrm{mV} & then & \left\{\begin{array}{c} v\leftarrow c\\ u\leftarrow u+d \end{array}\right. \end{array}$$

**Figure 1:**
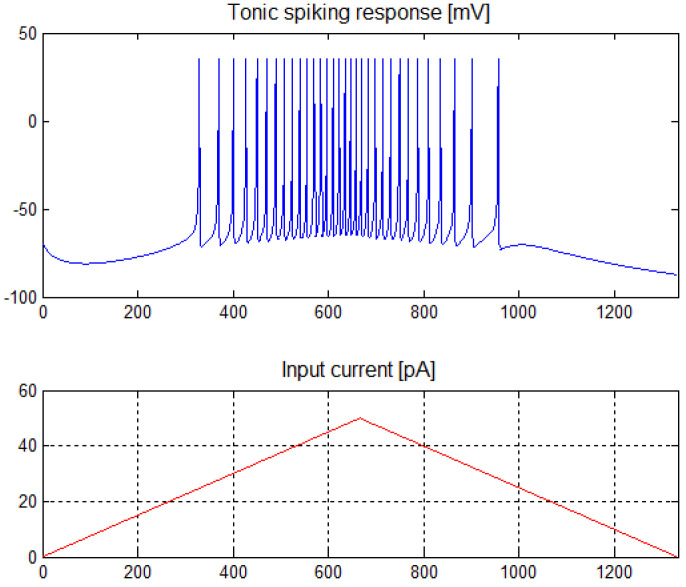
Tonic spiking neuron response for the input current in the range of 0 ÷ 50 pA.

In the above model, the variables *v* and *u* are calculated based on the value of input current $I_{app}$ and constants a, b, c, d, the values of which make it possible to select the type of neuron. As mentioned, we used two types of neurons in our approach. For tonic spiking neurons, the parameters had the following values: *a* = 0.02, *b* = −0.1, *c* = −65, *d* = 6. The characteristics of this neuron are shown in Figure 1. It is only valid for positive input currents.

**Figure 2:**
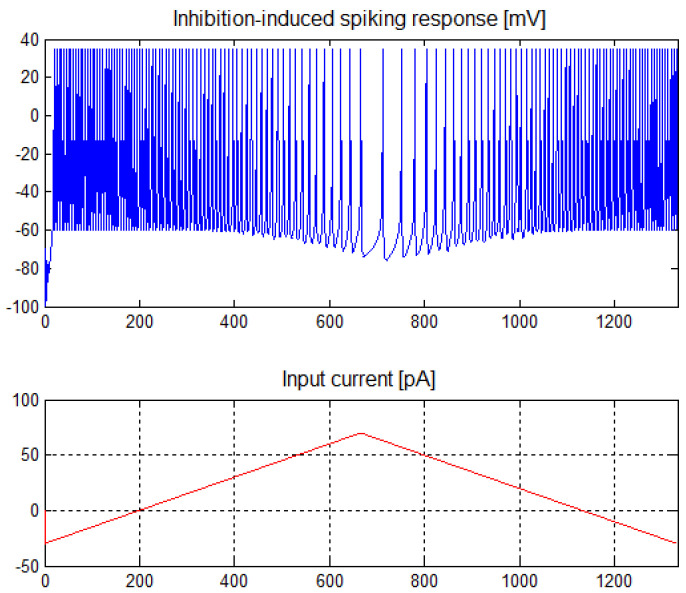
Inhibition-induced spiking neuron response for an input current of −30 ÷ 70 pA.

For inhibition-induced spiking neurons, these parameters have the following values: *a* = −0.02, *b* = −1, *c* = −60, *d* = 8. The characteristics of these neurons are shown in Figure 2. These neurons show complementary characteristics to the spiking tonic neurons. They work correctly in a wide range of positive currents, for which they show a lower frequency of generated spikes, and of negative currents, for which the frequency of the generated spikes increases. Due to the symmetrical polarization, in the case of inhibition-induced spiking neurons, it is acceptable to use negative weights in the input layer of the network with data standardized in the range of positive values.

### 2.2. Network Routing

**Figure 3:**
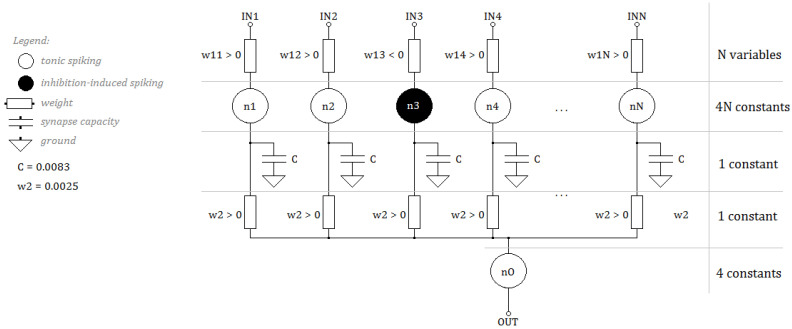
SNN architecture with linear computational complexity.

The aim of our research was to develop an SNN architecture guaranteeing high processing precision using the lowest possible algorithm complexity. Most networks processing data from sensors are multi-layer networks, the layers of which are fully connected, often with additional backward connections [16,17]. An often used solution, at the stage of selecting an architecture, is to define, apart from the input layer, a separate excitatory neuron layer and a separate inhibitory neuron layer [17,18,19]. The multi-layer architecture and the large number of connections between neurons are the source of the high complexity of the network algorithm. Taking into account the number of connections in multi-layer networks, we decided to give up the classic approach of using separate excitatory neuron layers and inhibitory neuron layers and limited ourselves to using tonic spiking and inhibition-induced spiking neurons in a common single layer. Thus, the backward connections were also omitted. We proposed the network architecture shown in Figure 3. It is a solution dedicated to time waveform analysis in the form of sensor data. Therefore, the input data are signals sampled with a certain frequency and sequenced in registers. The input layer size of *N* corresponds to the sampling of the analyzed signals. A single sample is fed to one of the neurons in the first layer of neurons n1−nN. The number of neurons in this layer corresponds to sampling and equals *N*. Similarly, the number of weights connecting the data layer to the first layer of neurons equals *N*. Two types of neurons may occur in the first layer: tonic spiking neurons marked in white in Figure 3 and inhibition-induced spiking neurons marked in black. The type of the neuron depends on the value of the weight that connects it to the network input.

A detailed algorithm for determining the values of weights w11−w1N is described in Section 3. The mapping process (selecting the type of neuron) is therefore a part of the network learning process. The network has an output layer, marked in Figure 3 as n0. This neuron has *N* inputs; each connects it to a single neuron in the preceding layer. It should be emphasized that all weights of these connections are positive and, above all, identical. This means that they do not change during the learning process. In the diagram, they are marked as w2. Using identical weights, i.e., the ones that guarantee zero dispersion, resulted in a significant reduction of the complexity of the hardware implementation. The value of the weights w2 equaled 0.0025 and was determined by simulating networks of different sizes for different sampling.

In connections between neurons, the authors used a mechanism for modeling synapse plasticity using a capacitor [20]. The current at the output of the synapse equaled the sum of resistance currents *R* of the synapse (reciprocal of its weight) and the capacitor charging current according to Equation (4). For all synapses, the value of capacitance $C_{ref}$ was the same and equaled 0.0083.

(4) $$I_{soma}=I_{Res}+I_{Cap}=\frac{V}{R}+\frac{dV}{dt}C_{ref}$$

The learning process required selecting *N* input weights, which become variables, and determining types of neurons in their first layer. Each neuron was defined with constants a, b, c,  d according to Equations (2) and (3). The constants for the neurons of the last layer were always the same as for the tonic spiking neuron and did not require mapping. Similarly, the weights and capacitances connecting layers of neurons did not require determination. Ultimately, the complexity of the network algorithm equaled: *N* variables and 4N+6 constants.

## 3. Network Learning

**Figure 4:**
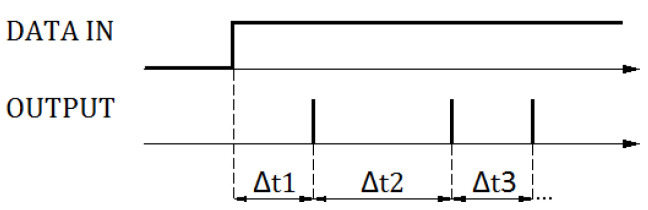
The applied SNN response coding.

The aim of the learning process was to determine the values of the input weights and to map the first layer of neurons using these weights. Due to the small number of variables, the authors did not use the method of Spike-Timing-Dependent Plasticity (STDP) [21], nor the classical optimization using, e.g., backpropagation for spiking neural networks [22]. These methods turned out to be ineffective and did not guarantee appropriate parameters of precision or the selectivity of patterns. On the other hand, the learning process used mechanisms similar to methods based on pseudo-inversion matrices or Hebbian methods, but with reference to associative memories, in which it is possible to determine weights analytically. Therefore, the classic approach of selecting weight values using iterative algorithms was not used. The coding method used was directly related to the applied learning algorithm. Due to the very small number of variables, the following methods turned out to be ineffective: time to first spike, resonant burst coding, coding by synchrony, phase coding. The authors used a modified coding method, namely latency coding based on the exact timing of spikes [23], shown in Figure 4, based on the analysis of the latency times between subsequent spikes of the response-producing neuron.

Due to learning without a teacher, latency times Δt1, Δt2, Δt3… were specific to patterns and did not require modification during the learning process. The number of latencies necessary for pattern analysis depended on the complexity of patterns and affected the specificity of the network algorithm. In practice, for the examples of time waveforms tested for network analysis, in order to obtain a precision above 0.96, it was sufficient to compare two latencies, i.e., the times of occurrence of the first two spikes.

The detailed workflow of the learning algorithm was defined as the pseudocode in Algorithm 1. The input data of the algorithm, except of learning set IN, were constants in the form of weight values of output layer wout and the capacitances Cref of synapses connecting layers. The input data also included parameters a, b, c, d defining tonic spiking (TS()) and inhibition-induced (IIS()) models and the constant map of connections in the network (SNN()), the size of which was the result of the size of patterns in set IN. The algorithm begins with the analysis of Set (1), i.e., determining the number of patterns and their sampling size. In case of IoT applications, the size of patterns is usually defined by the system architect. Steps (3)–(7) define the process of determining input weights using a simple linear function, out of the elements of the set according to the matrix Equation (5). Vector W1 contains all input weights, while the AVG vector stores the averaged values of the signal samples.

(5) W1=δ(κ−AVG)

It was required to select learning parameters δ and κ. Their values for standardized input currents are given in Section 4, with a description of an example of network learning concerning the problem of data analysis from a set containing samples of biomedical signals.

The loop that maps neurons of the first layer (Lines 8–12) assigns models of neurons depending on the sign of weight values. Such a criterion requires standardization of input currents in terms of positive values, so that the input current at tonic spiking neurons is always positive. Negative current values are still acceptable from the point of view of the neuron model, but are interpreted as a zero current, and the information they carry is lost. Weights of the subsequent layer (13) and synaptic capacitances (14) are assigned based on constants.

Latency times Δt1, Δt2 are determined as a mode of the SNN response for all elements of the learning set (16). For test set *M* of size *m*, the mean squared error of the network response is defined according to Equation (6).

(6) $$MSE=\frac{1}{m}\sum_{i=1}^{m}\left[{\left(\Delta t1-\Delta t1_{M(i)}\right)}^{2}+{\left(\Delta t2-\Delta t2_{M(i)}\right)}^{2}\right]$$

Output data of the learning algorithm are weights, capacitances, a neuron map, and latency.
**Algorithm 1** Network mapping and routing.**Require:** dataset IN; constant output weight wout; constant synapse capacitance Cref; neuron models IIS() and TS(); function of neural network SNN()**Ensure:** First layer weights w1; second layer weight w2; synapse capacitance *C*; first layer neuron map; linear function f(); response code Δt1 and Δt2  *Reading the set*:1:[sampling, patterns]=size(IN)2:**for**i=1 to sampling **do**3: **for** j=1 to patterns **do**4:  
L(i)←L(i)+IN(i,j)5: **end for**6: avg(i)←L(i)/patterns  *Calculation of input weights*:7: 
w1(i)←f(avg(i))8: **if** w1(i) < 0 **then**9:  
n(i)←IIS()10: **else**11:  
n(i)←TS()12: **end if**  *Assigning output weight*:13: w2(i)←wout  *Assigning synapse capacitance*:14: 
C(i)←Cref15:**end for** *Response coding*:16:[Δt1,Δt2]←mode(SNN(IN))17:**return**[w1,w2,C,n,Δt1,Δt2]

## 4. Pattern Classification

In this section, we present a practical application of an SNN with linear complexity in the processing task of edge computing of biomedical time waveforms. The section presents network parameters for different levels of signal sampling.

### 4.1. Current-Mode Signals

**Figure 5:**
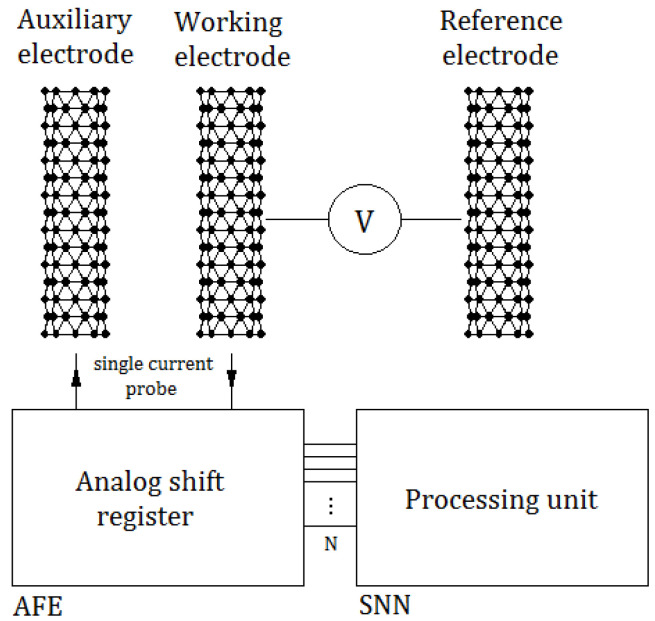
Application of the described SNN in the task of analyzing data from CNT sensors.

As an example of a practical application, the authors chose an amperometric system for use in monitoring vesicle release in the process of exocytosis [24]. The advantage of this measurement method is the acquisition of data in the form of current waveforms, which due to the current-voltage nature of the neuron model (Equation (1)) made it possible to directly process data from sensors in the SNN. Sensors in amperometry are usually electrodes implemented using Carbon NanoTubes (CNTs) [25], and the analyzed currents were in the range of zero to several dozen pA. The diagram of a waveform monitoring system using a neuroprocessor is shown in Figure 5.

The system uses a set of three electrodes: auxiliary, working, and reference electrodes. The AFE (Analog-Front-End) circuit is an analog shift register [26], to which single samples of current signals from electrodes are sent in discrete time. The output of the analog register is an analog data bus with a size corresponding to the sampling parameter used in Algorithm 1 and the network *N* size, as shown in Figure 3. The processing unit was implemented using the network presented in the above-mentioned diagram.

The detection of substances using amperometric techniques is a relatively simple operation requiring only the use of comparators. Waveform analysis, on the other hand, is a computational problem. An example of a computational task requiring the classification of amperometric waveforms is tracking processes of cell exocytosis based on vesicle fusion taking place inside cells. This approach is used in cancer metastases’ analysis [27] and early diagnostics of diseases such as Alzheimer’s [28], cholestasis [29], hypoxia [30], thrombosis [31], and tetanus [32]. The main problem is the need to analyze signals not coming from a single cell and a single set of CNT electrodes, but from tens of thousands of cells and a huge number of electrode sets. The hardware implementation of such a large number of neural networks requires using exceptionally simple and effective network algorithms. It is therefore an excellent example of a practical application of the approach described in this paper.

**Figure 6:**
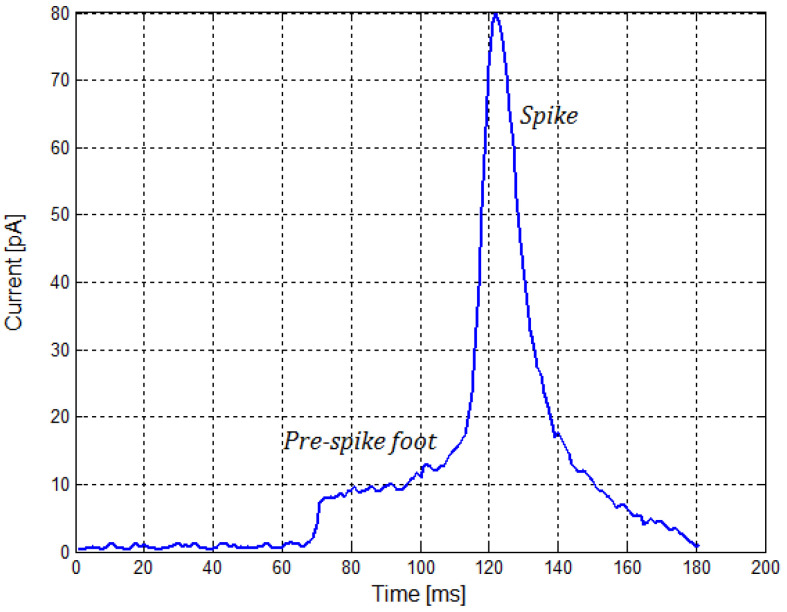
Amperometric waveform showing full vesicle fusion.

CNT electrodes for the above-mentioned applications are generally implemented as carbon nanotube arrays [33]. They make it possible to monitor current waveforms corresponding to vesicle fusion events inside a tissue. An example of a current waveform of the electrodes corresponding to a single fusion is shown in Figure 6. Current values made it possible to directly stimulate synaptic connections in the SNN using signals from the CNT electrodes.

It is worth mentioning, when discussing the current mode of network operation in the presynaptic area, that spiking neurons in hardware implementations are mainly found in the form of silicon structures designed using CMOS technologies [6,34,35,36]. The miniaturization of nanometer CMOS technologies forces the designers of such circuits to replace the voltage processing mode with the current mode [7,37]. An SNN application for amperometric techniques in the IoT is an excellent answer to the problem of the miniaturization of the technology for implementing modern neuroprocessors.

### 4.2. Classifier Efficiency

**Figure 7:**
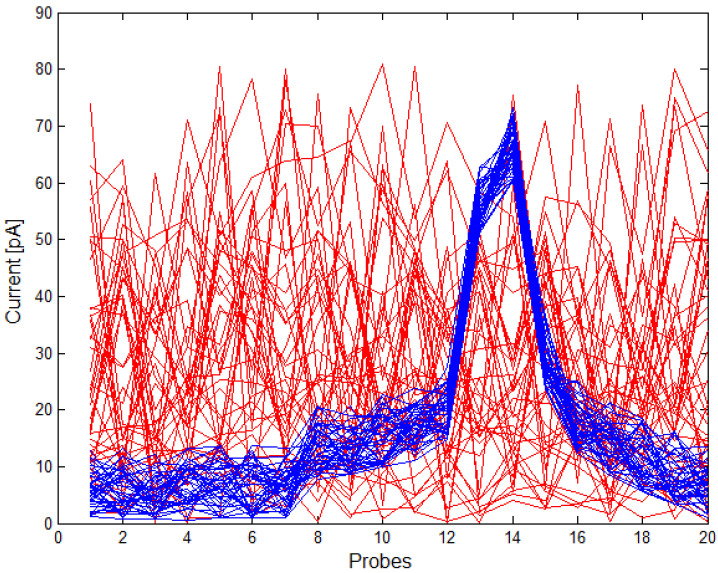
Positive — and negative — patterns used in SNN training with the sampling parameter N=20.

In this part, we present the results of an SNN implementation in the task of the classification of vesicle fusion waveforms for different waveform sampling frequencies and, thus, for different network sizes: 10-1, 15-1, 20-1, 25-1, 30-1, 35-1, 40-1. The analyses used set 40 positive patterns and 40 negative patterns. The patterns of positive and negative waveforms are presented in Figure 7.

The implementation details are described for the example of the network 20-1. The network input layer consisted of 17 tonic spiking neurons and three inhibition-induced spiking neurons. Weights for tonic spiking neurons were in the range of 8.08 ÷ 29.23 and for inhibition-induced neurons in the range of −55.71 ÷ −2.64. Learning parameters had the following values: δ=1.4,κ=26. Two-latency-based coding was used.

The result of the classifier 20-1 operation is presented in Table 1. The first part of the table shows network responses for positive patterns and the second part for negative patterns. In both parts, the patterns are ordered according to the increasing Squared Error (SE) they generated, calculated according to Equation (6) with m=1 in each case. The mode determined based on positive patterns equaled: Δt1 = 3 ms, Δt2 = 3 ms. For 21 positive patterns, the network response followed the mode; therefore, their SE error equaled zero. The remaining positive patterns generated a non-zero SE error with a maximum value of 10. For all negative patterns, this error was at least twice as large, as shown in Table 1. Since the error for all negative patterns was greater than the largest error for positive patterns, the accuracy equaled one. A decrease in accuracy may be only due to obtaining a smaller SE for at least one of the negative patterns, rather than the highest SE for positive patterns.

Table 2 lists network parameters of various sizes. Columns TS and IIS list the number of Tonic Spiking and Inhibition-Induced Spiking neurons, respectively. The subsequent columns present the analysis results of the following set: TP, True Positive; TN, True Negative; FP, False Positive; FN, False Negative. Accuracy was calculated based on these four values according to Equation (7). The accuracy for all architectures was higher than 0.96 and for most examples, especially the more complex ones, equaled one. It is worth emphasizing that the decrease in accuracy was not directly caused by the complexity of the network, but by too poor sampling for the cases 10-1 and 15-1. Thus, it is not a feature of the network itself, but of sampling of the signal using too low accuracy.

(7) $$\mathrm{ACC}=\frac{\mathrm{TP}+\mathrm{TN}}{\mathrm{TP}+\mathrm{FN}+\mathrm{FP}+\mathrm{TN}}$$

The last column presents the complexity of individual networks calculated based on the diagram in Figure 3 according to Equation (8), with (v-variable, c-constant). The complexity of the network was therefore linear, and the number of variables equaled exactly the size of the input data.

(8) COMPLEX=Nv+(4N+6)c

All examples used coding based on two latencies. The adopted coding method defines the maximum sampling frequency of the amperometric signal. The maximum sampling frequency is the reciprocal of the sum of the maximum latencies for the positive patterns and was 100 Hz for the analyzed example.

The values of parameters δ and κ were different for each network. Parameter δ was in the range of 0.95÷1.4, and parameter κ was in the range of 26÷32. There is no analytical method to determine them, but both parameters depend on the size of input layer *N* according to Equation (9).

(9) $$\kappa ∼N,\delta ∼\frac{1}{N}$$

In conclusion, the study featured a learning method based on the algorithm described using the Algorithm 1 pseudocode including dedicated coding. As mentioned at the beginning of Section 3, the traditional approach using common coding methods proved ineffective. Table 3 contains the parameters of a 20-1 architecture network obtained for different approaches: the detection time of a single fusion, i.e., the maximum generation time of the response for a positive pattern and the accuracy parameter. Some of the approaches made it possible to detect the positive pattern two times faster, but featured an accuracy below 0.8. Due to using a classifier in the analysis of slowly changing amperometric signals, it is more effective to use an approach that guarantees high precision, even with a longer analysis time. The most similar to the approach based on two latencies is counting time to first spike. The method described in the paper was an extension of this coding method with counting time to the next impulse, which made it possible to increase the level of precision from 0.95 to 1.0. According to Table 2, for the method based on two latencies, the accuracy was kept at the same level for all of the described architectures; whereas limiting oneself to a single impulse resulted in the following accuracies for the given architectures: 25-1: ACC = 0.978, 30-1: ACC = 0.956, 35-1: ACC = 0.961, 40-1: ACC = 0.966.

### 4.3. Mismatch Analysis

Finally, we would like to present the results of the analysis of the sensitivity of the discussed network to phenomena typical for the implementation of SNNs as semiconductor circuits. The basic problem of fabricating circuits in nanometer technologies is the mismatch of process parameters leading to a change in the thickness of the gate oxide in field-effect transistors [38]. As for SNNs, the change in threshold voltages of MOS transistors caused by this phenomenon leads to a change in scaling factors of the current mirror multipliers, and thus in the weights of connections in the network [39]. The mismatch problem is the basic source of damages to integrated circuits at the fabrication stage and requires proposing effective testing methods [40].

**Figure 8:**
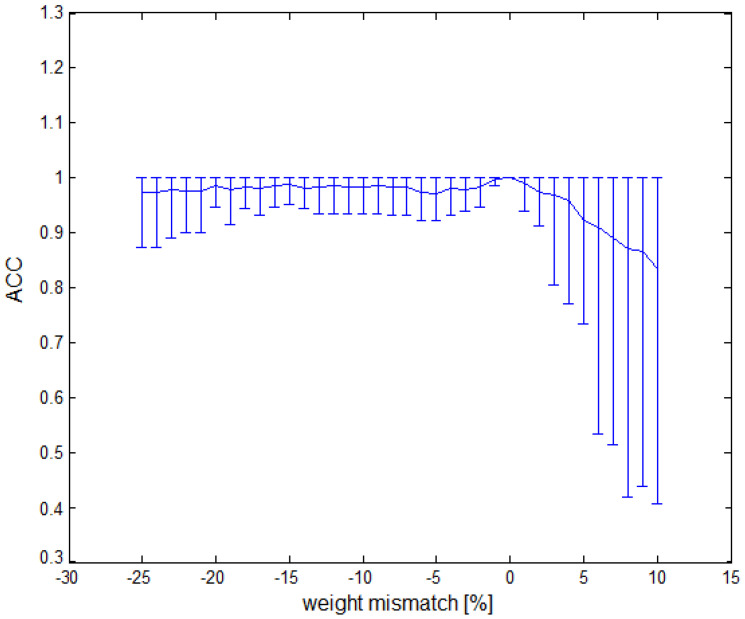
Accuracy vs. weight mismatch.

Due to changes in the values of weights in synaptic connections at the fabrication stage, we analyzed the degradation of the network response to a random dispersion of weights w11−w1N and w2 for the network 20-1 in detail. Results of the analysis are presented in Figure 8. The change in the thickness of the gate oxide in the process had a gradient character and was similar for transistors placed in the same substrate area. For this reason, the analysis was performed depending on the percentage error and separately for positive and negative errors. The analysis was performed for 50 random samples in each percentage range, and Figure 8 presents the trend of the mean value with error bars representing extreme values. What draws attention is the strong asymmetry in network sensitivity. While for negative dispersion, the network maintains accuracy at the level of 0.9 up to −20% changed in synaptic weights, the same level of accuracy was only maintained for 2% of positive dispersion. When planning a semiconductor network implementation, it is worth considering a 10% correction of values of weights w11−w1N and w2 in order to eliminate network sensitivity, especially as the network showed great tolerance for mismatch in the range of −20%÷2%. In the case of a software implementation or using an FPGA, this feature does not apply, unless, due to the reduction of weight values, it significantly reduces the bit representation of the performed operations.

## 5. Discussion

In the current section, we would like to conduct a cost analysis of a hardware implementation of the discussed network, which is dedicated to IoT problems. There are different structures of spiking neurons implemented using semiconductor technologies [6,35,36]. According to the best knowledge of the authors of this article, implementations of the lowest complexity require using six field-effect transistors and two capacitors [7] or 15 transistors without a capacitor [41]. As for the implementation of synapses, due to the current mode, the multiplication operations are performed using circuits with reconfigurable current mirrors [42], which also makes it possible to invert the current flow direction, and thus also the implementation of negative weights. The cost of implementing positive weights with a dispersion in the range of 0÷64.0 is 56 transistors and concerning negative weights in the range of −64.0÷0 is 60 transistors. The second layer weights, which connect neurons, due to the zero dispersion, can be implemented using mirrors without reconfiguration. The cost of implementing such synapses is four transistors. The cost of implementing the entire SNN 20-1 network with ACC = 1 described in the previous section was 1338 transistors and 62 capacitors in the case of CMOS neurons of type [7] or 1527 transistors and 20 capacitors in case of CMOS neurons of type [41]. For comparison, the implementation of a 1 bit digital multiplication operation requires using 48 transistors [43]. This means that a single weight implemented with a 32 bit precision requires using 1536 transistors, while the entire SNN was implemented using a similar amount of resources. We estimated the parameters of a semiconductor implementation of a network of type [41] with the 20-1 architecture. The analysis was performed using the TSMC 65 nm technology and the Eldo simulator. According to the estimation, the classifier’s power consumption equaled 146 nW. The energy consumption calculated for the whole network equaled 2.9 pJ/spike. The maximum operating frequency of the classifier was 1170 patterns/s with the coding shown in Table 1.

It is worth mentioning that ensuring zero dispersion of weights in connections between neurons is an introduction to a wider research of the authors on training SNNs. The results of these studies showed that the network kept all its parameters of precision, selectivity, specificity, and sensitivity, even with complete elimination of weights from its entire structure, i.e., using weights of the same value in all elements of the network. The results of these studies are at the publication stage. In this article, we wanted to draw attention to the specific insensitivity of the network to the value of weights and to the possibility of eliminating most connections in the network without compromising its parameters. Total reduction of weight spread in the second layer of the network, ensuring low network sensitivity to mismatch, and the reduction of the complexity of the network algorithm itself reduced the cost of calculations performed in sensors.

Reducing the network algorithm complexity is one of the more frequently discussed topics in literature. One of the hardware friendly architectures of SNNs was the implementation described in paper [17]. The paper presented the implementation of a neuroprocessor as a CMOS circuit. The network architecture consisted of an input layer, a fully connected excitatory neuron layer, and an inhibitory neuron layer with feed-forward and -backward connections. Despite the fabrication-oriented implementation, the network complexity was high due to the large number of connections. For this reason, seventy-eight-point-seven percent of the surface of the layout of the described neuroprocessor was occupied by synapses. The network was tested for patterns 14.4 times wider than in case of our network with the 40-1 architecture. However, the number of synapses in this case was 2875 times greater. Another implementation, which has a particularly low complexity, is the network described in paper [44] dedicated to processing black-and-white photos. The simplified architecture of the SNN, described in the paper, was focused on implementation using an FPGA, in which case, the number of available resources is very limited. The authors of the study put much emphasis on a strong reduction of the number of neurons in the network, yet the linear complexity of the network was not guaranteed. As the authors pointed out, the number of neurons must be increased in relation to the number of classes for the network to correctly classify patterns. A limitation was also the number of required 200 Time Units (TUs) for the correct classification of patterns. Providing the adopted refractory period of 30 TUs, the classification required generating six impulses. In our coding, we limited ourselves to two impulses, and analyses presented in Table 1 showed a correct classification of patterns already at 10 TUs. Thanks to such coding, we were able to obtain a 20 times faster pattern processing speed. The network described in paper [44] was used to analyze black-and-white patterns with a few percent of noise added or partially obscured. In our study, we analyzed analog signals changing in the entire range of acceptable values of network input currents. Thanks to this, the solution can be used in sensor techniques.

Lastly, the authors would like to point out potential limitations of the presented approach. The main disadvantage was using neurons based on the Izhikevich model, which admittedly are characterized by low complexity, yet the number of floating point operations in their case is greater compared to the integrate-and-fire model. Moreover, in order to ensure high precision in the classification task, the learning process featured an original method of determining weights instead of the commonly used methods. As a limitation of the described approach, it is also worth indicating the range of processed input currents. Passing data to the network directly from electrodes is possible only assuming that input currents do not exceed values typical for amperometry, i.e., 100 pA. Otherwise, it is required to use additional input current scaling circuits operating in current mode. Similarly, when it is necessary to analyze currents of smaller values, the input layer requires using weights with a larger dispersion of values.

## 6. Conclusions

This paper presented a waveform classifier based on spiking neural networks for applications in edge computing. We proposed a structure of a network based on the activity of the diencephalon and with linear computational complexity, in which the number of variables, i.e., connection weights, was significantly reduced, limiting it only to the number of processed samples. Additionally, we completely eliminated variables between network layers and replaced them with connections of identical values. Despite significant reductions in the weighting mechanism, analyses of networks of various sizes showed very good accuracy parameters, regardless of the size of the network. Additionally, the paper analyzed the cost of implementing the discussed structures as CMOS circuits.

## Figures and Tables

**Table 1:** Network answer.

	Patterns	Δt1 (ms)	Δt2 (ms)	SE
positive *	1	3.0	3.0	0
	2	3.0	3.0	0
	3	3.0	3.0	0
	…	…	…	…
	21	3.0	3.0	0
	22	4.0	5.0	5
	23	4.0	5.0	5
	…	…	…	…
	38	4.0	6.0	10
	39	4.0	6.0	10
	40	4.0	6.0	10
negative	1	5.0	7.0	20.0
	2	8.0	4.0	26.0
	3	8.0	5.0	29.0
	4	8.0	5.0	29.0
	5	8.0	5.0	29.0
	6	9.0	4.0	37.0
	…	…	…	…
	36	18.0	15.0	369.0
	37	18.0	15.0	369.0
	38	19.0	15.0	400.0
	39	25.0	13.0	584.0
	40	27.0	8.0	601.0
* mode = [3.0, 3.0].

**Table 2:** Comparison of network parameters of different sizes.

SNN	TS	IIS	TP	TN	FP	FN	ACC	COMPLEX
40-1	35	5	40	40	0	0	1	40v + 166c
35-1	31	4	40	40	0	0	1	35v + 146c
30-1	26	4	40	40	0	0	1	30v + 126c
25-1	22	3	40	40	0	0	1	25v + 106c
20-1	17	3	40	40	0	0	1	20v + 86c
15-1	13	2	40	38	2	0	0.975	15v + 66c
10-1	9	1	40	37	3	0	0.9625	10v + 46c

**Table 3:** Comparison of the 20-1 network parameters vs. coding methods.

	Period (ms)	ACC
resonant burst coding	10	0.783
coding by synchrony	5	0.798
phase coding	12	0.941
time to first spike	4	0.946
two latencies	10	1

## Data Availability

No new data were created or analyzed in this study. Data sharing is not applicable to this article.

## Abbreviations

The following abbreviations are used in this manuscript:

SNN	Spiking Neural Network
CNT	Carbon NanoTube
CMOS	Complementary Metal Oxide Semiconductor
ADC	Analog-to-Digital Converter
FPGA	Field-Programmable Gate Array
GPU	Graphics Processing Unit
STDP	Spike Timing-Dependent-Plasticity
TS	Tonic Spiking
IIS	Inhibition-Induced Spiking
AFE	Analog Front-End
SE	Square Error
TP	True Positive
TN	True Negative
FP	False Positive
FN	False Negative
TUs	Time Units
